# The Odor of the White Race

**Published:** 1903-09

**Authors:** 


### THE ODOR OF THE WHITE RACE.

It may not be generally known that the European races have an odor as peculiar and as characteristic as the African. Though to themselves not perceptible, to non-Europeans it is as conspicuous as that of the negro is to us. A Japanese, Buntaro Arachi, writing in *Globus*, describes his impressions of it. (Das Geruch der Europäer.) The odor of the European is generally known in Japan. To the Japanese the odor of the European, especially that of the young women, is very striking. It is pungent and rancid, differing somewhat with the individual, sometimes sweetish or bitter. The odor is often so strong that a room is filled with it. It is closely connected with the age of the person. Children and old people either do not smell, or smell less than those of stronger age. It may easily be believed that the European is not aware of his odor, or is at least less aware of it than the Japanese. It is very certain, however, that the European does not know that the odor is peculiar to him, and it is equally certain that he has not made especial comment on it. As a rule men find the odor of women and their environment more agreeable than their own. It is interesting to note that the Japanese finds that the odor of European women changes after a time. When he has resided for some time in Europe he finds the odor of the women of the country at first very disagreeable, after a time, however, it becomes less so, and finally may even be agreeable. In his opinion the odor is undoubtedly dependent upon sexual activity. The odor proceeds from the axillary spaces, which are so hairy that, except in nearly scentless individuals, thorough washing with soap does not remove it, and if it does do so it rapidly returns. The odorous material originates from the sweat glands. In regard to the axillary odor being associated with the sexual function it may be remembered that in certain animals during the rutting season the function of the odoriferous glands is unusually active.

As concerns the racial odor of the yellow race Adachi does not find any general characteristic as among whites and negroes. It is occasionally, but rarely, observed among the Japanese, and then chiefly among women. Yekishiu, or, in the vernacular, Waki Kusa ("armpit odor"), is identical with that of the European. According to Chinese medical books this odor is seldom observed among that race. A Japanese the subject of Yeki-Shiu is exempt from military duty, and if this odor is emitted by a Japanese young lady her matrimonial chances are decidedly diminished. Adachi thinks that after marriage the odor appears to be less objectionable because, perhaps, the connubial partner finds it, from its association, not unenjoyable, though of course he can bring forward no proof of this opinion. Ordinarily the axillary spaces of the Japanese have no odor perceptible either to themselves or to Europeans, even after prolonged abstention from bathing.

It seems to Adachi that Europeans sweat more than the Japanese, as he has observed that the clothing in the axillary region in Europeans is easily sweated through, a thing very seldom seen among the Japanese. It is an undoubted and conspicuous fact that the sweat glands of the European people are very much larger than in the Japanese, among whom they are not to be found macroscopically. The odor of the Europeans cannot, however, be attributed solely to the sweat function, as among profusely sweating Japanese there is no axillary effluvium.

Without dealing with the microscopic orientation of the axillary gland in contrast to the Japanese, Adachi declares that the sweat glands of the European are larger, and that his armpits are malodorous.

